# Deciphering the Relationship Between Astrocyte Calcium Signaling, Neurovascular Coupling, and Cerebrovascular Function in Alzheimer’s Disease

**DOI:** 10.1002/alz.095554

**Published:** 2025-01-09

**Authors:** Blaine E. Weiss, John C Gant, Ruei‐Lung Lin, Susan D Kraner, Olivier Thibault, Pradoldej Sompol, Christopher M. Norris

**Affiliations:** ^1^ University of Kentucky College of Medicine, Lexington, KY USA; ^2^ University of Kentucky, Lexington, KY USA; ^3^ University of Kentucky College of Medicine, Sanders‐Brown Center on Aging, Lexington, KY USA

## Abstract

**Background:**

Astrocytes are a glial cell type responsible for many protective functions in the brain. While they are primarily recognized for regulating synaptic activity, they’re also essential for maintaining the neurovascular unit, and cerebral hyperperfusion during metabolic demand. Calcium signaling has been identified as a regulatory process for these functions. Amyloid Beta deposits (Aβ) are a primary diagnostic marker of Alzheimer’s disease, and are linked to synaptic degeneration, astrocyte reactivity, and cognitive decline. Alzheimer’s disease also frequently presents with vascular damage and hypoperfusion, suggesting impaired astrocyte function. We investigated the impact of amyloid burden on stimulation‐evoked vasoreactivity and astrocyte calcium signaling to determine potential patterns of physiological impairment that may contribute to Alzheimer’s disease pathology.

**Methods:**

Six‐month‐old 5XFAD and littermate control mice were injected with AAV2/5‐Gfa104‐jGCaMP8f into barrel cortex and imaged three weeks later, while awake, using two‐photon microscopy. Neurovascular coupling experiments were conducted using timed air puff stimulation of whiskers, and calcium signals were recorded from activated astrocytes. Calcium transient properties were analyzed over different cellular compartments by custom developed Matlab applications. Vascular tone in response to stimulation was also measured, and correlations calculated with endfeet signaling.

**Results:**

Astrocytes from 5XFAD mice showed a significant reduction in calcium signaling amplitudes, (F(1,53) = 8.735, p = 0.0047, n = 25,32) compared to wild type controls with a significant deficit in female 5XFAD mice. Correlations between other signaling properties such as rise/decay kinetics, and network connectivity were also characterized between the 5XFAD and control mice. Astrocyte endfoot compartments also showed reduced transient amplitudes. (F(1,30) = 3.226, p = 0.033, n = 17,15) Neurovascular coupling in the 5XFAD mice was reduced (F(1,39) = 2.511, p = 0.015, n = 20,19) despite no changes in arteriole elasticity. A correlation between astrocyte calcium signaling and the magnitude of stimulation‐induced vasodilation was observed in wild‐type mice but not in the 5XFAD group.

**Conclusion:**

Amyloid induced pathology impairs the brain’s adaptivity to neuronal stimuli at the neurovascular unit. The uncoupling between vasoreactivity and astrocyte signaling processes implies that amyloid accumulation may render the brain vulnerable to conditions of neuronal hyperexcitability and metabolic dysregulation observed in Alzheimer’s disease.

